# Cleanliness of the Teeth

**Published:** 1839-08

**Authors:** C. A. Harris


### CLEANLINESS OF THE TEETH,

*Its importance to their health and preservation, and the means necessary to its accomplishment.*

[ BY C. A. HARRIS, M. D. ]

The importance of a proper attention to the cleanliness of the teeth, is to the profession too well known to need comment; the remarks, therefore, which we design making upon the subject, will be intended more for the benefit of the general reader, than for that of the practitioner of dentistry; and to him it would seem almost superfluous to speak particularly, were we not apprized of the great diversity of opinion that exists among those who have never made the diseases of these organs a matter of much enquiry. By the educated and refined, its utility is pretty generally acknowledged; yet there are a few, even among this description of persons, who regard it as decidedly injurious, and as an evidence of the correctness of their views, we are referred to individuals fifty, sixty, or perhaps even seventy or eighty years of age, whose teeth are perfectly sound, that have never paid any attention to their cleanliness; and to others, not more than eighteen or twenty, that were known to be frequent and regular in its observance, with deplorably bad ones. To a person unacquainted with the differences in the liability of the teeth of different individuals to become diseased and the causes that produce them, this might seem to be a very conclusive argument; but to one having a knowledge of them, it only proves, that in proportion as these organs are hard or soft; that their power or capability of resisting morbid impressions is increased or diminished. Hence, teeth, that are very hard, rarely decay; whereas, those that are soft decay from the most trivial causes; and thus it is, that some occasionally retain them to an advanced period of life, while others, and that too, very frequently, lose them before they arrive at any thing like what is usually denominated middle age.

It often happens, however, that persons who are possessed of the hardest kind of teeth, lose them early in life by the destruction of the means of their support; and it is worthy of remark, that the loss of the teeth in this way is generally confined to those who pay little or no attention to their cleanliness. With those who are, and have been from early childhood, in the frequent and regular observance of this, such an occurrence rarely if ever happens; and the reason is obvious:—By cleaning the teeth regularly, two or three times a day, the formation of tartar is not only prevented, but such particles of food and other extraneous matter as lodge about and adhere to them, and which, when permitted to remain, soon become putrid, vitiating the juices of the mouth, and causing them to irritate and inflame the gums, and in consequence, to be absorbed, together with the alveoli, are also removed. That there is a greater tendency in some than others to disease of the gums, we do not deny; but this, by proper care and attention might, we believe, in almost every instance, be counteracted. Our opinion, therefore, is, that the gums, if the teeth be kept *perfectly* clean, seldom become spongy or recede from their necks; and that teeth, that are situated in healthy gums, rarely, if ever, loosen much in their sockets.

If this, then, be true—and that it is we are firmly pursuaded—the importance of a strict observance of the purifying of the mouth, cannot, if for no other purpose than the keeping of the gums healthy, and the prevention of a consequent loosening of the teeth, be too strongly urged.

As a prophylactic against diseases of the teeth themselves, or at least of their crowns, it is also powerful—we might, perhaps, say certain—for, were it possible to keep them perfectly clean, we believe they would never decay. But this, literally speaking, is hardly practicable. The enamel on the sides of the teeth that come in contact with each other, is often fractured, and within these fractures, and the indentations on the grinding surfaces of the bicuspides and molares, a lodgment is afforded to small particles of extraneous matter, and the secretions of the mouth that cannot, by any of the ordinary purifying means, be reached, and thereby remaining there until vitiated and putrid, exercise upon the parts with which they are in contact, an exceedingly hurtful influence. But were it carried as far as it is practicable, the teeth would be much less frequently affected with disease; in fact, we do not believe there would be more than one decayed tooth, where there are now half a dozen; and in this opinion we are convinced, that every one whose attention has been much directed to the subject, will fully concur. It is one to which we have paid much attention; and the result of our observations is, that those who do, and have from early youth attended to the cleaning of their teeth frequently, regularly and thoroughly, have altogether and by far better ones than those who have neglected it, or who, if they have attended to it at all, have done it at irregular and uncertain intervals. This we have noticed in families, and in those parts where the members resembled each other in features, constitutional temperament, and in the shape, size, colour and arrangement of their teeth, and our enquiries in almost every instance, have tended to the confirmation of this opinion. Nor could it be otherwise; for upon the presence of putrid foreign matter and vitiated mucus and saliva, does most of the diseases of these organs depend.

The fact, then, that cleanlines of the teeth is essential to their health and preservation being established, a motive sufficiently strong for its observance is no longer wanting. But were this not the case, others, though comparatively of much less weight—but which with many may have a greater influence—might be urged. Of these, the principal and only ones which we at this time propose to notice, are personal appearance and comfort, and upon them we shall offer but a few remarks.

To a pleasing and agreeable expression of the countenance, a clean and healthy denture is of the greatest consequence. Even if the teeth lack that *perfect* regularity of arrangement which it is desirable that they should have, if they be but free from tartar, viscid mucus and extraneous matter, their appearance will never be disagreeable; but if any of them, and more especially those occupying the anterior part of the mouth, be discolonred or covered with clammy mucus or tartar, or any other kind of foreign matter, they will rarely fail, however perfect all the other features of the face may be, to give to the countenance an unpleasing aspect.

However scrupulously particular persons of either sex may be in regard to their dress, and although they may be decked with jewels, and sparkle with diamonds, if they at the same time be unmindful of a proper attention to the cleanliness of their teeth, they will fail to excite in individuals of refined taste, those feelings of admiration which most desire to elicit, and which these organs when the requisite care has been bestowed upon them always attract.

And as essential as this is to personal appearance, it is equally so to comfort; and this of itself ought to be a sufficient inducement to the regular observance of such means as are necessary to its accomplishment; but so remiss are some persons in regard to it, that they allow their teeth to be constantly covered with putrid extraneous matter, and their breath, in consequence, to be so offensive as to render their proximity to another a source of the greatest annoyance. The frequent and thorough cleaning of these organs is of the greatest consequence to a pure breath, and those who neglect it, breathe a more or less contaminated air, which, although it may not be obvious to themselves, is very perceptible to others. It is also requisite to health, for in proportion as the teeth are unclean, especially those of persons who have delicate constitutions, is the appetite impaired and the digestive organs weakened; and thus the capability of enjoying life is often greatly diminished. But to enter into an explanation of the manner in which the health of the general system becomes affected by the want of a proper attention to the teeth, constitutes no part of our present purpose; it may, however, from what has been said in regard to its effects upon the organs themselves, and upon the gums and alveolar processes, be very readily inferred.

Having now shown the importance to the health and preservation of the teeth, of keeping them constantly clean, we shall proceed to describe the manner by which it is to be effected, premising, that if they or the parts within which they are contained, be diseased, such means as are necessary to their restoration to health, should first be resorted t.o This done, all that will usually be required to keep them clean, will be a tooth-pick, a brush, and pure water. The first of these should be constructed from a goose-quill, and should always be used immediately after eating, that such particles of food as may have gotten between the teeth, may be removed before they shall have undergone a chemical change. As it regards the kind of brush most proper to be employed, there exists a considerable diversity of opinion ; some prefer a very hard one, others think a brush cannot be too soft; whilst others again, give their preference to a medium one, and to this last our own is decidedly inclined. A tooth brush should be neither too hard nor *too* soft, but of these qualities the last is least objectionable, and for a child is perhaps preferable to any other. The bristles should be about one half of an inch in length, and be elastic, so that they may be made to reach and act upon all the inequalities of the teeth, and with such a brush as this, and water, they should be thoroughly rubbed several times a day : say, for instance, in the morning immediately after getting up, and after every meal. This purifying process should be commenced in early childhood, and if it be carried to a sufficient extent, it will prevent the teeth from becoming stained or discoloured, and keep the gums healthy.

Dentifrices, such as powders, lotion, &c. are only called for in the treatment of diseased teeth and gums, therefore, of them we shall say nothing at present.

